# Receptor tyrosine kinases play a significant role in human oligodendrocyte inflammation and cell death associated with the Lyme disease bacterium *Borrelia burgdorferi*

**DOI:** 10.1186/s12974-017-0883-9

**Published:** 2017-05-30

**Authors:** Geetha Parthasarathy, Mario T. Philipp

**Affiliations:** Division of Bacteriology and Parasitology, Tulane National Primate Research Center, Tulane University, 18703, Three Rivers Road, Covington, LA 70433 USA

**Keywords:** *B. burgdorferi*, Human oligodendrocytes, Inflammation, Receptor tyrosine kinases, Lyme neuroborreliosis

## Abstract

**Background:**

In previous studies, human oligodendrocytes were demonstrated to undergo apoptosis in the presence of *Borrelia burgdorferi* under an inflammatory milieu. Subsequently, we determined that the MEK/ERK pathway played a significant role in triggering downstream inflammation as well as apoptosis. However, the identity of receptors triggered by exposure to *B. burgdorferi* and initiating signaling events was unknown.

**Methods:**

In this study, we explored the role of several TLR and EGFR/FGFR/PDGFR tyrosine kinase pathways in inducing inflammation in the presence of *B. burgdorferi*, using siRNA and/or inhibitors, in MO3.13 human oligodendrocytes. Cell death and apoptosis assays were also carried out in the presence or absence of specific receptor inhibitors along with the bacteria to determine the role of these receptors in apoptosis induction. The expression pattern of specific receptors with or without *B. burgdorferi* was also determined.

**Results:**

TLRs 2 and 5 had a minimal role in inducing inflammation, particularly IL-6 production. Rather, their effect was mostly inhibitory, with TLR2 downregulation significantly upregulating CXCL8, and CXCL (1,2,3) levels, and TLR5 likely having a similar role in CXCL8, CXCL(1,2,3), and CCL5 levels. TLR4 contributed mostly towards CCL5 production. On the other hand, inhibition of all three EGF/FGF/PDGF receptors significantly downregulated all five of the inflammatory mediators tested even in the presence of *B. burgdorferi*. Their inhibition also downregulated overall cell death and apoptosis levels. The expression pattern of these receptors, as assessed by immunohistochemistry indicated that the PDGFRβ receptor was the most predominantly expressed receptor, followed by FGFR, although no significant differences were discernible between presence and absence of bacteria. Interestingly, inhibition of individual EGFR, FGFR, or PDGFR receptors did not indicate an individual role for any of these receptors in the overall downregulation of pathogenesis. Contrarily, suppression of FGFR signaling alone in the presence of bacteria significantly upregulated inflammatory mediator levels indicating that it might control an inhibitory pathway when triggered individually.

**Conclusions:**

Unlike TLRs, EGF/FGF/PDGF receptors collectively play a significant role in the inflammation and apoptosis of human oligodendrocytes as mediated by *B. burgdorferi*. It is likely that these three receptors need to be triggered simultaneously to achieve this effect.

## Background

Lyme neuroborreliosis (LNB), caused by the spirochete *Borrelia burgdorferi*, is a morbid form of Lyme disease with complex neurological sequelae. LNB manifests in about 15% of patients with primary erythema migrans, and may be evidenced as a lymphocytic meningo-radiculitis, lymphocytic meningitis, cranial neuritis, radiculoneuritis, radiculoneuropathy, and/or, rarely, encephalitis and myelitis [1]. The pathogenesis of LNB is not clearly understood. We have established a rhesus macaque model and several in vitro and ex vivo models to help delineate mechanisms of acute LNB. Intrathecal inoculation of live *B. burgdorferi* in rhesus macaques elicited in these animals signs of acute LNB, including leptomeningitis, radiculitis, inflammatory lesions in dorsal root ganglia (DRG), accompanied by glial and neuronal apoptosis in the DRG [2]. Stereotaxic intraparenchymal inoculations in rhesus macaques have similarly shown oligodendrocyte and neuronal cell death by apoptosis in the central nervous system (CNS) [3]. In either study, apoptosis of neuroglial cells occurred in an inflammatory environment, leading us to hypothesize that inflammation elicited by *B. burgdorferi* is a leading cause of LNB pathogenesis. In proof of principle, administration of dexamethasone, an anti-inflammatory agent, in vivo in rhesus macaques, not only downregulated the pleocytosis of lymphocytes and monocytes in the cerebrospinal fluid (CSF) but also glial and neuronal cell death [4]. In separate in vitro studies, *B. burgdorferi* has been documented to induce inflammation and cell death by apoptosis in neurons in the presence of microglia [5], and without any other cell involvement in the case of oligodendrocytes [6]. Downregulation of inflammation by pretreatment with dexamethasone, also inhibited oligodendrocyte cell death. In addition, a study of the mechanisms of inflammation in oligodendrocytes has shown that the MEK/ERK pathway plays a predominant role in inducing not only the production of several key inflammatory mediators but also the expression of the transcription factor p53 [7]. Inhibition of the ERK pathway suppressed p53 levels, and suppression of either the MEK/ERK pathway or the mitochondrial p53 downregulated apoptosis in oligodendrocytes, indicating that this mitogen-activated protein kinase (MAPK) pathway plays an important role in both inflammation and apoptosis of these glial cells. In continuation of this study, the current manuscript explores the roles of several surface receptors in mediating such downstream events, and we show that the signaling mechanisms in human oligodendrocytes in response to *B. burgdorferi* in vitro are part of a multi-factorial complex process with non-traditional receptors playing key roles.

## Methods

### Bacterial strain and culture

*B. burgdorferi* B31 5A19, a strain possessing the full complement of plasmids [8] was used throughout this study. The strain was routinely cultured in Barbour-Stoenner-Kelly (BSK-H) medium (Sigma Aldrich, St. Louis-MO) supplemented with amphotericin (0.25 μg/mL), phosphomycin (193 μg/mL), and rifampicin (45.4 μg/mL), for about 5–6 days, under microaerophilic conditions. Concentration of bacteria was determined using a dark-field microscope, and the required number was harvested by centrifugation at 2095×*g* for 30 min at room temperature, without brakes. The resulting bacterial pellet was resuspended in DMEM high glucose (Invitrogen/Life Technologies, Inc., Grand Island-NY) supplemented with 100 nM phorbol myristate acetate (PMA) (Sigma Aldrich, St. Louis-MO) to the same concentration prior to pelleting and diluted further to the required multiplicity of infection (MOI).

### Cell culture

The human oligodendrocyte cell line MO3.13 (CELLutions Biosystems Inc., Ontario, Canada) was used to model the effects of *B. burgdorferi* on glial cells and cultured as described in Parthasarathy and Philipp [7]. MO3.13 cells are adult human oligodendrocytes fused with human rhabdomyosarcoma cells. Cells routinely grown in DMEM (high glucose) supplemented with 10% fetal bovine serum (FBS) and 1% penicillin/streptomycin (P/S) at 37 °C, 5% CO_2_, were seeded on 6-well plates (0.8 × 10^4^/well), or chamber slides (0.6 × 10^4^/well). After 3 days, cells were allowed to differentiate into mature oligodendrocytes by replacing the culture medium with DMEM high glucose devoid of serum but supplemented with 100 nM PMA and 1% P/S. Cells were allowed to differentiate for 3 days further before experiments were carried out.

### Infection assays with receptor inhibitors

The differentiated MO3.13 cells were exposed to *B. burgdorferi* at an MOI of 10:1 for 48 h. The experimental medium was identical to the differentiation medium but without antibiotics. At 2 h prior to the addition of bacteria, receptor inhibitors or their solvent controls were added, to determine the role of several receptors on *B. burgdorferi*-induced inflammation in human oligodendrocytes. The following inhibitors were used: PD-089828-epidermal growth factor/fibroblast growth factor/platelet derived growth factor-receptor tyrosine kinase (EGF/FGF/PDGF RTK) inhibitor; AG-1478 (EGFR inhibitor); CP-673451 (PDGFRα/β inhibitor); PD 166866-FGFR inhibitor; SU6656 (src family kinase inhibitor); Oxidized 1-palmitoyl-2-arachidonoyl-sn-glycero-3-phosphorylcholine (OxPAPC) (TLR2/4); CLI-095 (TLR4); neutralizing antibody to human TLR5 and its isotype control. All of the growth factor receptor inhibitors except CP-673451 were from EMD Millipore (Billerica, MA), while the PDGFRα/β inhibitor was obtained from Selleck Chemicals (Houston, TX). The TLR inhibitors and antibodies were obtained from Invivogen, San Diego, CA. At 48 h after the addition of bacteria, supernatants were collected after centrifugation at 2095×*g*, 10 min, 4 °C to remove bacteria and cellular debris, aliquoted and stored at −20 °C until analysis by enzyme-linked immunosorbent assay (ELISA) for chemokines and cytokines.

### Ribonucleic acid interference (RNAi)

The role of TLRs in inducing inflammation in oligodendrocytes, in response to *B. burgdorferi* exposure was determined by gene silencing using small interfering RNA (siRNA) technology. Transfection complexes were generated using 2 μL HiPerfect transfection reagent (Qiagen, Valencia, CA) and 12.5–25 nM siRNA (Santa Cruz Biotechnology, Dallas, TX) in experimental medium. The complexes were incubated at room temperature for 30 min, and 200 μL of the complex was added to cells, after replacing the old medium in the 6-well plates. Immediately after the addition of transfection complexes, 800 μL of experimental medium was added to prevent drying of cell layer. After a 24-h incubation at 37 °C, 5% CO_2_, *B. burgdorferi* (MOI 10:1) was added and further incubated for 48 h, at the end of which, supernatants were collected as before and analyzed for chemokine and cytokine expression. A non-specific control siRNA was used as negative control for all experiments. Pam3CSK4 (Imgenex, San Diego, CA; TLR2), lipopolysaccharide (LPS) O55:B5 (Sigma Aldrich, St. Louis, MO; TLR4) were used as TLR-specific positive controls when required.

### ELISAs for chemokines and cytokines

The supernatants of glial cells from various experimental conditions were analyzed for specific chemokines and cytokines using custom Milliplex kits from EMD Millipore (Billerica, MA). The Multiplex ELISAs were carried out at the Pathogen Detection and Quantification Core, Tulane National Primate Research Center according to the manufacturer’s protocols.

### Cytotoxicity assay

The toxic effect of various inhibitor and siRNA concentrations on MO3.13 cell viability, if any, was determined using tetrazolium dye MTT, and as described in [7]. Viability of cells due to various treatments was normalized or compared to that of their respective controls (control siRNA, solvent or medium only controls).

### Immunohistochemistry

MO3.13 cells were immunostained for PDGFRβ and FGFR1-3 according to previously described protocols [7]. Briefly, cells growing in chamber slides (seeded at 0.6 × 10^4^ cells/well) were exposed to medium alone or *B. burgdorferi* at an MOI of 10:1 for 48 h. Following treatments, supernatants were removed, and cells were fixed in 2% paraformaldehyde for 10 min, followed by brief washes in phosphate-buffered saline (PBS). The cells were then permeabilized with an ethanol:acetic acid (2:1) mix, washed in PBS again, and kept in PBS at 4 °C until analysis by immunostaining. Cells were then re-permeabilized in PBS containing 0.1% Triton-X-100, blocked with 10% normal goat serum (NGS) for 1 h, followed by staining for PDGFRβ/FGFR1-3 with each specific primary anti-human rabbit polyclonal antibody (1:50; Santa Cruz Biotechnology, Dallas, TX) and a secondary goat anti-rabbit IgG antibody conjugated to Alexa Fluor 488 (1:800; Invitrogen) in PBS containing 10% NGS for 45 min–1 h each. EGFR staining was also carried out using a mouse monoclonal antibody (1:50; Santa Cruz Biotechnology, Dallas, TX) and a corresponding secondary antibody conjugated to Alexa Fluor 488 (1:800; Invitrogen). The nuclei were stained with TOPRO-3 iodide (1:1000; Invitrogen), when required. A negative control with only the secondary antibody, as well as myelin basic protein (MBP) staining as a positive control for cell type was carried out for each experiment. Slides were mounted with an anti-quenching reagent, covered with coverslips, and visualized for various receptor expressions.

### Terminal deoxynucleotidyl transferase dUTP nick-end labeling (TUNEL) assay

The effect of inhibitors on apoptosis of MO3.13 oligodendrocytes in the presence of *B. burgdorferi* was determined by TUNEL assay. After exposure of cells to *B. burgdorferi* in the presence or absence of inhibitor or medium control for 48 h, MO3.13 oligodendrocyte cells were fixed as described above, stained for oligodendrocyte-specific MBP, and the TUNEL assay was performed thereafter following the manufacturer’s instructions (EMD Millipore Apoptag Fluorescein kit) and as described in Ramesh et al. [6]. An anti-human MBP rabbit polyclonal was used as the primary antibody (1:100; Millipore) while the secondary antibody was a goat anti-rabbit IgG conjugated to Alexa Fluor 568 (1:1000; Invitrogen). At the end of the assay, the slides were mounted with an anti-quenching reagent and cover-slipped and visualized under a fluorescent microscope.

### Microscopy

A Leica DMRE fluorescent microscope (Leica microsystems, Buffalo Grove-IL) and Lumecor SOLA GUI software (Lumencor, Beaverton-OR) were employed for microscopy. Cells were imaged using Nuance Multispectral Imaging System (CRi, PerkinElmer, Waltham-MA), and Adobe® Photoshop CS6 software was used to assemble the images. For the TUNEL assay, to quantify differences between treatment groups, approximately 1000 cells were counted over 10 fields or more for each well along with TUNEL-positive cells in each field/frame. Percent apoptosis for each treatment was calculated as total TUNEL-positive cells/total cells × 100 and graphed using Microsoft Excel® software.

### Statistics

The Student’s *t* test was used to determine the statistical significance of an experimental outcome, with each analysis using duplicate values. The results were considered significantly different if the probability values (*p*) were <0.05.

## Results

### TLRs play a minor role in the induction of inflammatory responses in human oligodendrocytes

In our previous studies with microglia, TLRs 2, 4, and 5 were significantly upregulated in the presence of *B. burgdorferi* [9]. In follow-up studies, TLR2 and TLR5 were demonstrated to have a significant role in the induction of inflammatory responses in primary rhesus microglia due to exposure to *B. burgdorferi* [10]. Therefore, the roles of TLRs 2, 4, and 5 in similarly mediating an immune response to *B. burgdorferi* in MO3.13 oligodendrocytes were tested with the help of siRNA and inhibitors. Contrary to the results seen with microglia, TLRs only had a minimal role in inducing oligodendrocyte inflammation in the presence of the Lyme disease bacterium. At non-toxic concentrations of siRNA (toxic at levels higher than those described), suppression of TLR2 and TLR5 transcripts only affected interleukin 6 (IL-6) levels, while TLR4 suppression similarly affected (C-C) motif chemokine ligand 5 (CCL5) output (Table 1), modestly downregulating them, at 48 h post exposure to *B. burgdorferi*. There was rather a significant *increase* in output levels of CXCL8 and CXCL(1,2,3) after TLR2 mRNA suppression in the presence of *B. burgdorferi*, while TLR5 siRNA had a similar effect on CXCL8, CXCL(1,2,3), as well as CCL5. Other than a moderate downregulatory effect on CCL5 production, TLR4 mRNA suppression by RNAi did not appreciably affect other inflammatory mediators. To obtain additional evidence for such an effect, specific inhibitors or neutralizing antibodies were employed. In the presence of OxPAPC, a TLR2/4 inhibitor and *B. burgdorferi*, there was a significant increase in the production of CXCL8, CCL5, and CXCL(1,2,3) (Table 2), recapturing some of the effects of TLR2 siRNA. The TLR4-specific inhibitor CLI095 had a significant effect in downregulating CCL5 production in the presence of *B. burgdorferi*, again mimicking the effect of TLR4 siRNA. This inhibitor additionally downregulated CCL2 production while significantly increasing CXCL8 output levels. To obtain additional lines of evidence for TLR5-mediated effects, a human TLR5 neutralizing antibody was used. At 20 μg, the TLR5 neutralizing antibody in the presence of *B. burgdorferi* significantly upregulated CXCL8, CCL5, and CXCL(1,2,3) compared to induced levels by *B. burgdorferi* alone, in three independent experiments; similar to TLR5 siRNA effects. However, these levels were not significantly different from those obtained using *B. burgdorferi* with isotype control antibody (data not shown). As no specific TLR5 inhibitor is available, we are currently unable to corroborate the TLR5 siRNA data.

**Table 1:** Average fold change^a^ in chemokine/cytokine levels in the presence of specific siRNA at 48 h

Chemokine-cytokine/treatment	CCL2	CXCL8	CCL5	CXCL(1,2,3)	IL-6
*Bb* + 12.5 nM TLR2 siRNA	1.120^b^	*0.789*	1.051^b^	*0.883*	**1.362**
	(±0.368)	(±0.112)	(±0.176)	(±0.078)	(±0.264)
*Bb* + 12.5 nM TLR5 siRNA	0.769	*0.685*	*0.489*	*0.810*	**1.164**
	(±0.085)	(±0.032)	(±0.039)	(±0.024)	(±0.159)
*Bb* + 25 nM TLR4 siRNA	0.969	1.026	**1.050**	1.011	1.027^b^
	(±0.195)	(±0.078)	(±0.060	(±0.052)	(±0.169)

^a^Fold change was calculated as *B. burgdorferi* (MOI 10:1) + control siRNA/*B. burgdorferi* + TLR siRNA for each experiment. Average fold-change values were calculated from the mean of three independent experiments, with standard error of the mean within parenthesis. Fold change values >1 indicate a downregulation of chemokine/cytokine levels, and values <1 indicate an increase in those levels. Values in italics or those in bold indicate statistically significant upregulation or downregulation respectively, in a majority (>67% or 2/3) of experiments

^b^Inconclusive results

**Table 2:** Average fold change^a^ in chemokine/cytokine levels in the presence of TLR inhibitors at 48 h

Chemokine-cytokine/treatment	CCL2	CXCL8	CCL5	CXCL(1,2,3)	IL-6
*Bb* + 20 μg/mL TLR2/4 inhibitor	1.489 (±0.384)	*0.456* (±0.043)	*0.752* (±0.151)	*0.573* (±0.092)	0.915 (±0.092)
*Bb* + 5 μg/mL TLR4 inhibitor	**1.551** (±0.138)	*0.915* (±0.050)	**3.911** (±0.769)	0.993 (±0.062)	1.016^b^ (±0.104)

^a^Fold change was calculated as *B. burgdorferi* (MOI 10:1) + solvent control/*B. burgdorferi* + inhibitor for each experiment. In the case of the TLR2/4 inhibitor (OxPAPC), average fold change values were calculated from the mean of two to three independent experiments, while for the TLR4 inhibitor the values were calculated from the mean of three to four independent experiments. Standard error of the mean is indicated within parenthesis. Fold change values >1 indicate a downregulation of chemokine/cytokine levels, and values <1 indicate an increase in those levels. Values in italics or those in bold indicate statistically significant upregulation or downregulation respectively, in a majority (≥67%) of experiments

^b^Inconclusive results

In order to verify the siRNA and inhibitor efficacy as mediated through TLR2 and TLR4, appropriate positive controls were utilized. Unlike *B. burgdorferi*, which mediates upregulation of all five of the mediators tested, the TLR4 and TLR2 positive controls do not. LPS from O55:B5 (500 ng/mL) mediated upregulation of CCL2, CXCL8, and CCL5 from these cells compared to medium controls. Higher concentrations of LPS were toxic to cells. In the presence of 12.5 nM TLR4 siRNA, it significantly downregulated CCL2 and CXCL8, and in the presence of 5 μg/ml CLI-095, it significantly downregulated CCL2, CCL5, and had inconclusive effects on CXCL8. Similarly, Pam3Csk4 at 20 ng/mL, induced only IL-6 production when compared to medium controls. Higher concentrations of Pam3Csk4 were progressively inhibitory, and levels of chemokines and cytokines were mostly lower than that induced by 20 ng/mL concentration. In the presence of 12.5 nM TLR2 siRNA and Pam3Csk4 (20 ng/mL), IL-6 levels were significantly downregulated, along with CCL5 levels, while CXCL8 levels were significantly upregulated. In the presence of TLR2/4 inhibitor, the effect was upregulation of most of the inflammatory mediators, similar to the effects seen with *B. burgdorferi* (data not shown).

### Role of specific receptor tyrosine kinases (RTK) in inflammatory mediator output from human oligodendroglial cells

As TLRs did not show a marked effect in mediating inflammatory molecule production in MO3.13 oligodendrocyte cells in the presence of *B. burgdorferi*, the possible involvement of receptor tyrosine kinases such as EGFR and PDGFR was investigated. These receptors have been demonstrated to play a role in neuroinflammation associated with neuronal injury and neurodegenerative diseases such as multiple sclerosis and others [11–14]. In the presence of *B. burgdorferi* and various concentrations of an inhibitor that prevents signaling through EGFR/FGFR/PDGFR, there was a dose-dependent downregulation of various chemokines and cytokines (Table 3). At 1 μM, this RTK inhibitor significantly downregulated CCL2 levels, while significantly increasing CXCL(1,2,3) and IL-6 levels. However at 5 μM, there was a statistically significant downregulation of four of the five inflammatory mediator levels with the exception of CCL5. At even higher doses, at 10 μM, the inhibitor in the presence of *B. burgdorferi* significantly suppressed production of the five mediators, indicating that these receptors play a role in MO3.13 inflammatory output as mediated by *B. burgdorferi*. Interestingly, in a previous study, we reported that ERK inhibition (with 10 μM U0126) in the presence of *B. burgdorferi* downregulated CCL2, CXCL8, CXCL(1,2,3), CCL5, and IL-6 levels by 18.1-, 6.4-, 1.8-, 2.1-, and 5.9-fold respectively [7], comparable for the most part to the values achieved with the RTK inhibitor reported here.

**Table 3:** Average fold change^a^ in chemokine/cytokine levels in the presence of EGF/FGF/PDGF RTK inhibitor at 48 h

Chemokine-cytokine/treatment	CCL2	CXCL8	CCL5	CXCL(1,2,3)	IL-6
*Bb* + 10 μM inhibitor	**15.097** (±6.803)	**3.134** (±0.189)	**1.234** (±0.160)	**1.415** (±0.162)	**1.602** (±0.491)
*Bb* + 5 μM inhibitor	**15.677** (±5.388)	**3.270** (±0.672)	1.000 (±0.138)	**1.313** (±0.207)	**1.562** (±0.456)
*Bb* + 1 μM inhibitor	**4.094** (±0.597)	1.103 (±0.014)	1.432 (±0.268)	*0.646* (±0.034)	*0.665* (±0.153)

^a^Fold change was calculated as *B. burgdorferi* (MOI 10:1) + solvent control/*B. burgdorferi* + inhibitor for each experiment. Average fold change values were calculated from mean of three independent experiments, with standard error of the mean within parenthesis. Fold change values >1 indicate a downregulation of chemokine/cytokine levels, and values <1 indicate an increase in those levels. Values in italics or those in bold indicate statistically significant upregulation or downregulation respectively, in a majority (>67% or 2/3) of experiments

### Role of EGF/FGF/PDGF RTK signaling in *B. burgdorferi*-mediated apoptosis of oligodendrocytes

**Fig. 1:**
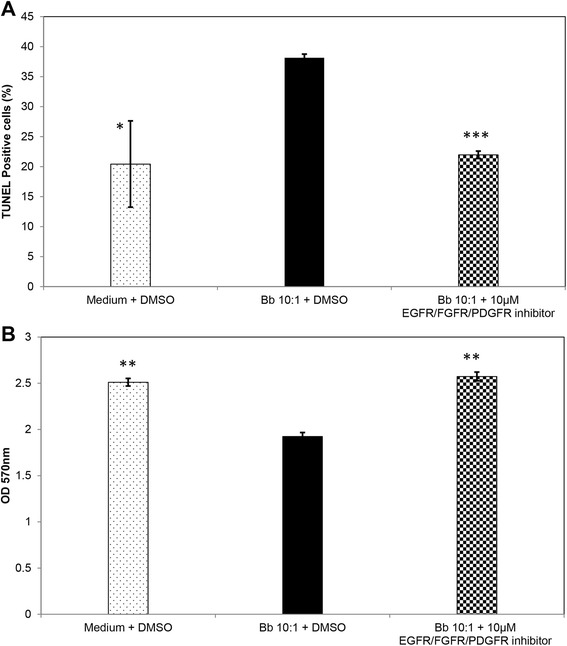
RTK inhibitor downregulates cell death associated with *B. burgdorferi* exposure. Differentiated MO3.13 cells were assessed for cell death via apoptosis and total cell viability in the presence or absence of *B. burgdorferi* (*Bb*, MOI 10:1) and 10 μM EGFR/FGFR/PDGFR tyrosine kinase inhibitor. DMSO was added as a solvent control. **a** TUNEL-positive cells at 48-h post exposure. In **b**, cell viability as assessed by the MTT assay at 72-h post exposure is shown. Values from a representative experiment are shown in each case (out of three experiments for **a** and two experiments for **b**). *Bars* represent standard deviation. *p < 0.05; **p < 0.01; ***p < 0.001

In our previous studies, we have demonstrated that inflammation has a causal role in apoptosis of oligodendrocytes, as application of dexamethasone, an anti-inflammatory agent downregulated not only inflammation as expected but also apoptosis when used in the presence of *B. burgdorferi* [6]. As the RTK inhibitor significantly downregulated all of the chemokines and cytokines tested, in the presence of *B. burgdorferi*, we next investigated its role in MO3.13 cell death as mediated by the bacterium. As seen in Fig. 1a, in the presence of 10 μM inhibitor, the percentage of TUNEL-positive cells was significantly diminished even with the bacteria present, to a value that was close to that induced by medium alone. Similarly, when total cell viability was tested through an MTT-based assay, the viability of cells in the presence of *B. burgdorferi* significantly improved when the inhibitor was added (Fig. 1b). This indicated that signaling through these receptors not only mediates inflammation in MO3.13 oligodendrocytes but also cell death as induced by *B. burgdorferi*.

### Expression of tyrosine kinase-containing receptors in MO3.13 oligodendrocytes and their individual role in inflammatory mediator production

**Fig. 2:**
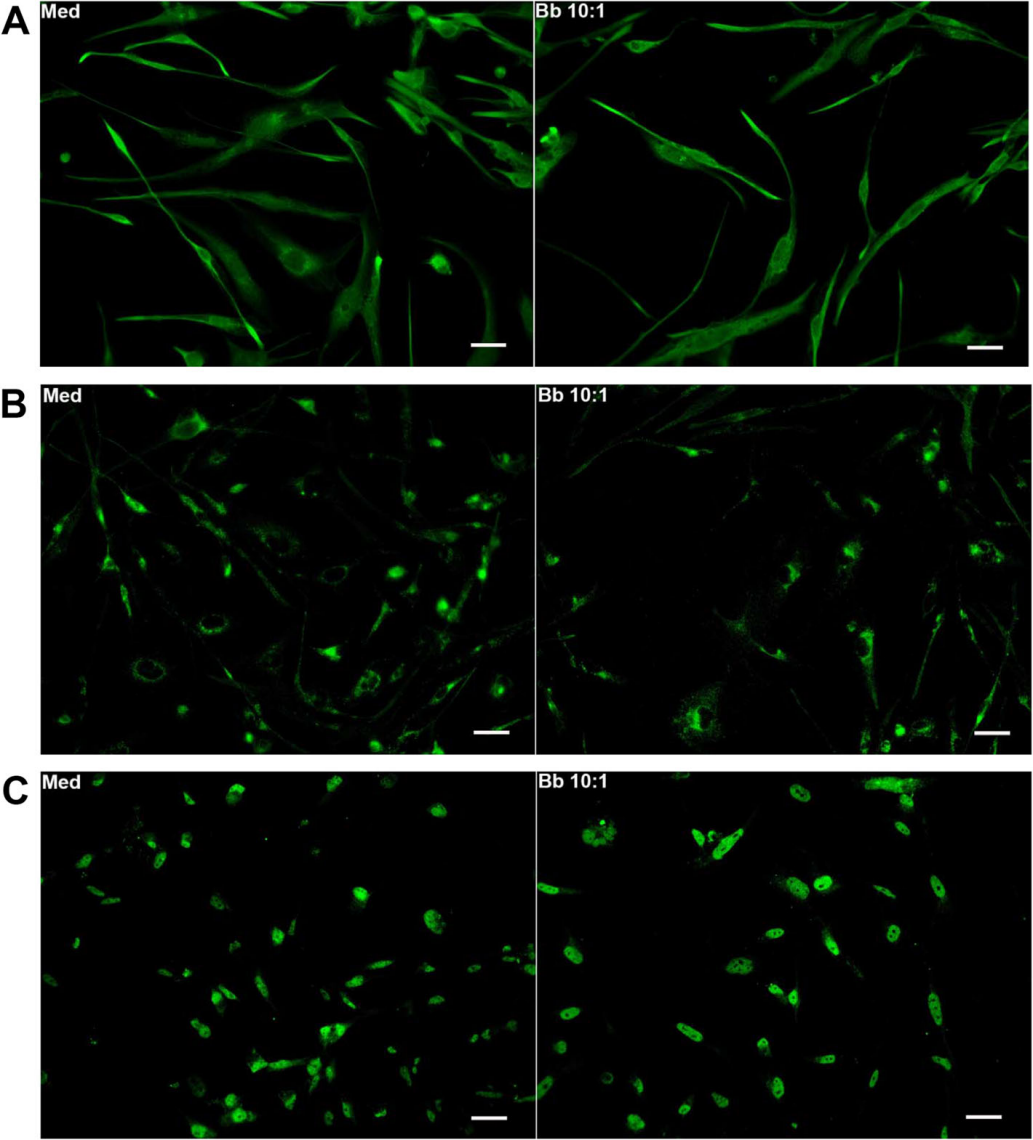
Differentiated human oligodendrocytes express RTKs. Immunofluorescence staining of MO3.13 cells for PDGFRβ (**a**), FGFR1 (**b**), and FGFR3 (**c**) is shown. Differentiated MO3.13 cells growing in chamber slides were exposed to either medium alone (*Med*) or *B. burgdorferi* (*Bb*, MOI 10:1) for 48 h, fixed with paraformaldehyde as described in the “Methods” section, and assessed for various receptors’ expression. Cells were additionally stained for MBP as a positive control, while the secondary antibody alone was used as a negative control (not shown). *Bar* represents 50 nm. A representative photograph from three to four experiments for each receptor is shown

As these receptors proved to have a marked effect on inflammation and cell death in human oligodendrocytes as mediated by *B. burgdorferi*, we next investigated their individual cellular expression in the presence or absence of the bacterium as well as their individual role in inflammation. PDGFRβ expression was seen throughout the differentiated cells with no obvious qualitative or quantitative differences in expression in the presence or absence of bacteria, as determined by immunohistochemistry (Fig. 2a). FGFR1 expression was evident around the nuclear region (Fig. 2b), although it was also present in some cytoplasmic areas. FGFR3 expression was seen mostly in the nuclear region (Fig. 2c). TOPRO staining, conducted separately, confirmed this phenotype for FGFR1 and FGFR3 (not shown). As with PDGFRβ, we did not see any significant difference in expression between medium and *B. burgdorferi-*treated cells through this staining assay. Incubation with a monoclonal antibody to EGFR did not yield a visible signal in a preliminary study, while the FGFR2 expression pattern was not conclusive (not shown). Overall, PDGFRβ was the most abundant, followed by the FGFRs.

Although the inhibitor primarily affects signaling associated with EGFR/FGFR and PDGFR, according to the manufacturer it also inhibits c-src, an intracellular signaling molecule that may or may not be receptor associated. Therefore, we next looked at the individual roles of all of these molecules in inducing inflammation from oligodendrocyte cells as mediated by *B. burgdorferi*. As seen in Table 4, suppression of each receptor alone did not identify a trigger for the effects described in Table 3. In contrast, with the exception of the PDGFR inhibitor, which downregulated CCL2 production, the individual inhibition of receptors increased the levels of chemokines and cytokines, with the most profound increase elicited by the FGFR inhibitor (higher concentrations of PDGFR inhibitor were toxic). Suppression of the src family of kinases at 5 μM inhibitor concentration did downregulate CCL2, CXCL8, and IL-6 levels significantly, indicating that this molecule might be an important factor in mediating inflammatory molecule release from MO3.13 cells in response to *B. burgdorferi*. An effort to determine the role of c-src in cell death via apoptosis and overall cell viability did not yield conclusive results (not shown).

**Table 4:** Average fold change^a^ in chemokine/cytokine levels with EGFR, FGFR, PDGFR, and src family kinase inhibitors

Chemokine-cytokine/treatment	CCL2	CXCL8	CCL5	CXCL(1,2,3)	IL-6
*Bb* + 10 μM EGFR inhibitor	1.466^b^ (±0.330)	*0.515* (±0.068)	*0.674* (±0.118)	0.657^b^ (±0.133)	0.836^b^ (±0.296)
*Bb* + 100 nM PDGFRα/β inhibitor	**1.733** (±0.461	*0.797* (±0.174)	0.977 (±0.123)	*0.738* (±0.124)	*0.478* (±0.048)
*Bb* + 5 μM FGFR inhibitor	*0.920* (±0.125)	*0.504* (±0.019)	*0.911* (±0.077)	*0.425* (±0.028)	*0.461* (±0.047)
*Bb* + 1 μM FGFR inhibitor	0.753 (±0.165)	*0.670* (±0.047)	*0.573* (±0.081)	*0.652* (±0.043)	*0.706* (±0.082)
*Bb* + 500 nM FGFR inhibitor	0.756 (±0.054)	*0.693* (±0.028)	*0.760* (±0.190)	*0.697* (±0.041)	*0.634* (±0.027)
*Bb* + 5 μM src family inhibitor	**1.184** (±0.168)	**1.258** (±0.079)	1.051 (±0.064)	0.964 (±0.022)	**1.465** (±0.080)
*Bb* + 1 μM src family inhibitor	0.901 (±0.173)	0.993^b^ (±0.081)	*0.778* (±0.125)	1.109 (±0.091)	1.035 (±0.082)
*Bb* + 500 nM src family inhibitor	0.893 (±0.122)	0.919 (±0.079)	0.847 (±0.036)	1.061 (±0.100)	1.044 (±0.137)

^a^Fold change was calculated as *B. burgdorferi* (MOI 10:1) + solvent control/*Bn burgdorferi* + inhibitor for each experiment at 48 h post exposure. Average fold change values were calculated from the mean of two to three independent experiments, with standard error of the mean within parenthesis. Fold change values >1 indicate a downregulation of chemokine/cytokine levels, and values <1 indicate an increase in those levels. Values in italics or those in bold indicate statistically significant upregulation or downregulation respectively, in a majority (2/2 or 2/3) of experiments

^**b**^Inconclusive results

## Discussion

Lipoproteins and flagellin are major components of *B. burgdorferi* that can initiate signaling events through TLR2 and TLR5, respectively, in many cell types [10, 15, 16], with TLR2-mediated events being the most widely studied. With respect to Lyme neuroborreliosis, TLR2, TLR4, and TLR5 expression was shown to be upregulated in primary rhesus microglia upon *B. burgdorferi* exposure [9] with TLR2 and TLR5 subsequently shown to play a significant role in the induction of immune responses to the Lyme disease bacterium in these glial cells [10]. Primary rhesus astrocytes showed an inflammatory response to L-OspA, a major TLR2-binding protein of *B. burgdorferi* as well as to FliC, a TLR5-binding ligand [9]. Hence, we decided to investigate whether these receptors similarly played a significant role in induction of inflammation in human oligodendrocytes, which were previously demonstrated to undergo apoptosis under an inflammatory milieu upon *B. burgdorferi* exposure [2, 6]. We show here that unlike with microglia or astrocytes, where these TLRs had a significant role in the induction of innate immune responses, oligodendrocyte TLR2 (and TLR5) had mainly an inhibitory role, with a modest role in IL-6 induction (Tables 1 and 2). TLR4 also had only a minor role in the induction of CCL5 as confirmed with both TLR4 siRNA and inhibitor. This was evidence to suggest that other receptors, either other TLRs, integrins, or novel receptors, likely had a significant role in the innate immune responses of oligodendrocytes to *B. burgdorferi*. It also indicated that although receptors such as TLR2 or TLR5 are common factors in *B. burgdorferi*-mediated signaling events in multiple cell types, differences do exist, and every cell type needs to be evaluated before a consensus can be reached, or treatment targets devised.

We next decided to look at non-TLR receptors that might play a pivotal role in mediating inflammation and apoptosis. In other studies, EGFR, PDGFR, and other receptor tyrosine kinases were shown to mediate neuroinflammation in various models of neurological disorders [11–13]. We found that inhibition of signaling through EGFR/FGFR/PDGFR downregulated not only the inflammation associated with *B. burgdorferi* exposure but also the accompanying cell death via apoptosis (Table 3 and Fig. 1). This indicated that in MO3.13 human oligodendrocytes, these specific receptor tyrosine kinases play a key role in the neuroinflammation and apoptosis that is associated with exposure to *B. burgdorferi*, similar to TLR2- and TLR5-mediated neuroinflammation in microglia [10].

In our previous work, we demonstrated that the ERK pathway plays an important role in both MO3.13 oligodendrocyte neuroinflammation and apoptosis via mitochondrial p53 [7]. In this study, we show that EGFR/FGFR/PDGFR-mediated signaling similarly directs both phenomena upon *B. burgdorferi* exposure. As these receptors are known to trigger the ERK pathway [17–20], it is likely that *B. burgdorferi* exposure triggers activation of these receptors, which in turn stimulates the ERK MAPK pathway predominantly, leading to inflammatory molecule production and p53-mediated apoptosis in these cells. We also undertook to investigate the role of these receptors in primary human oligodendrocyte cells (ScienCell Laboratories Carlsbad, CA), which have also been characterized with respect to inflammation, apoptosis, and the role of the MEK/ERK pathway in Lyme neuroborreliosis [6, 7]. Unfortunately, the concentrations of the pertinent reagents that were used with MO3.13 cells proved too toxic for the primary cells (data not shown). Due to the rare availability of these cells, currently, we are unable to continue experiments with primary human cells. We plan to resume this approach once the cells become available.

The RTK inhibitor, according to the manufacturer, inhibits EGFR, FGFR1, and PDGFRβ receptors. We next looked at these receptors’ expression pattern in order to discern any variability in expression upon *B. burgdorferi* exposure in comparison to cells containing medium alone. We did not see any visible signal for EGFR in a pilot study using a mouse monoclonal antibody. It is possible that this was a technical problem as all other antibodies used were polyclonal. EGFR expression has also been documented to decrease from embryonic to adult stages in the mouse midbrain, with higher expression in oligodendrocyte precursors. It is possible that this might be a factor wherein the expression was punctate and drowned out by background fluorescence [21]. Additionally, it is also possible that a mutated/truncated EGFR is present, not clearly detectable by the monoclonal. PDGF has been shown to increase myelin gene expression and number of mature oligodendrocytes [22]. As PDGF signals through its receptor PDGFR, it is not surprising to see very high expression of PDGFRβ in the mature MO3.13 cells (PDGFRα expression has been shown to be primarily in oligodendrocyte progenitors and pro-oligodendrocytes and absent in mature cells [23]). FGFRs particularly 1 and 2 are required for the generation of oligodendrocyte precursors [24], and FGFR3 for the terminal differentiation of oligodendrocytes [25], with their expression pattern controlled over the developmental stages. Although their levels vary, all three receptor forms are expressed by mature oligodendrocytes in rats [26]. The expression patterns of FGFR1 and FGFR3 are similar to those shown in some previously published studies [27–30]. FGFR2 was also expressed in these cells; however, the pattern of expression was not consistent, which might be attributed to technical issues with the antibody (not shown). Also, we could not establish significant differences in expression levels between medium control and those exposed to *B. burgdorferi* through receptor staining (Fig. 2). It is possible that differences might be established by staining for phosphorylated forms of the receptors or through Western blots.

We next attempted to delineate which of the three receptors involved primarily mediate(s) the neuroinflammation and cell death associated with *B. burgdorferi*. However, as seen in Table 4, the individual inhibition of each of these receptors did not elicit a major effect on suppression of multiple chemokines/cytokines. Neither did the combination of EGFR/FGFR, EGFR/PDGFR, or FGFR/PDGFR (data not shown). It is possible that inhibition of signaling through one receptor is compensated with increased signaling through the other two (or others) resulting in the increased production of inflammatory mediators as we observed (Table 4). Therefore, it is likely that all three receptors need to be inhibited simultaneously to achieve the multiple chemokines/cytokines suppression phenotype.

C-src, an intracellular tyrosine kinase, is upstream of ERK activation but below the RTKs in signaling sequence of events. It has been known to be associated with RTK such as EGFR, FGFR, and PDGFR among others [31]. While src kinase family inhibition had some significant effect on chemokine and cytokine production (Table 4), it did not achieve the level of inhibition seen with ERK inhibition alone [7]. It also did not produce conclusive effects on cell death (not shown). It is likely that another molecule associated with receptors, like phospholipase Cγ for instance [32] is also needed to initiate downstream events. Therefore, a cumulative contribution may be needed to achieve a threshold of ERK activation so as to generate the level of inflammatory output and p53 levels to initiate apoptosis. In view of these results, our tentative conclusion is that exposure of MO3.13 human oligodendrocytes to *B. burgdorferi* engages TLR2, 4, 5, EGFR/FGFR/PDGFRs, with the latter three being the main inducers of all five chemokines and cytokine; TLR2 and 5 perhaps contributing towards IL-6 output, while TLR4 effecting CCL5 production. On this basis, we hypothesize that activation of RTKs recruits c-src and others to their intracellular domains leading to increased activation of the MEK/ERK pathway via multiple receptors, yielding increased levels of inflammatory mediators and p53 levels to initiate apoptosis.

**Fig. 3:**
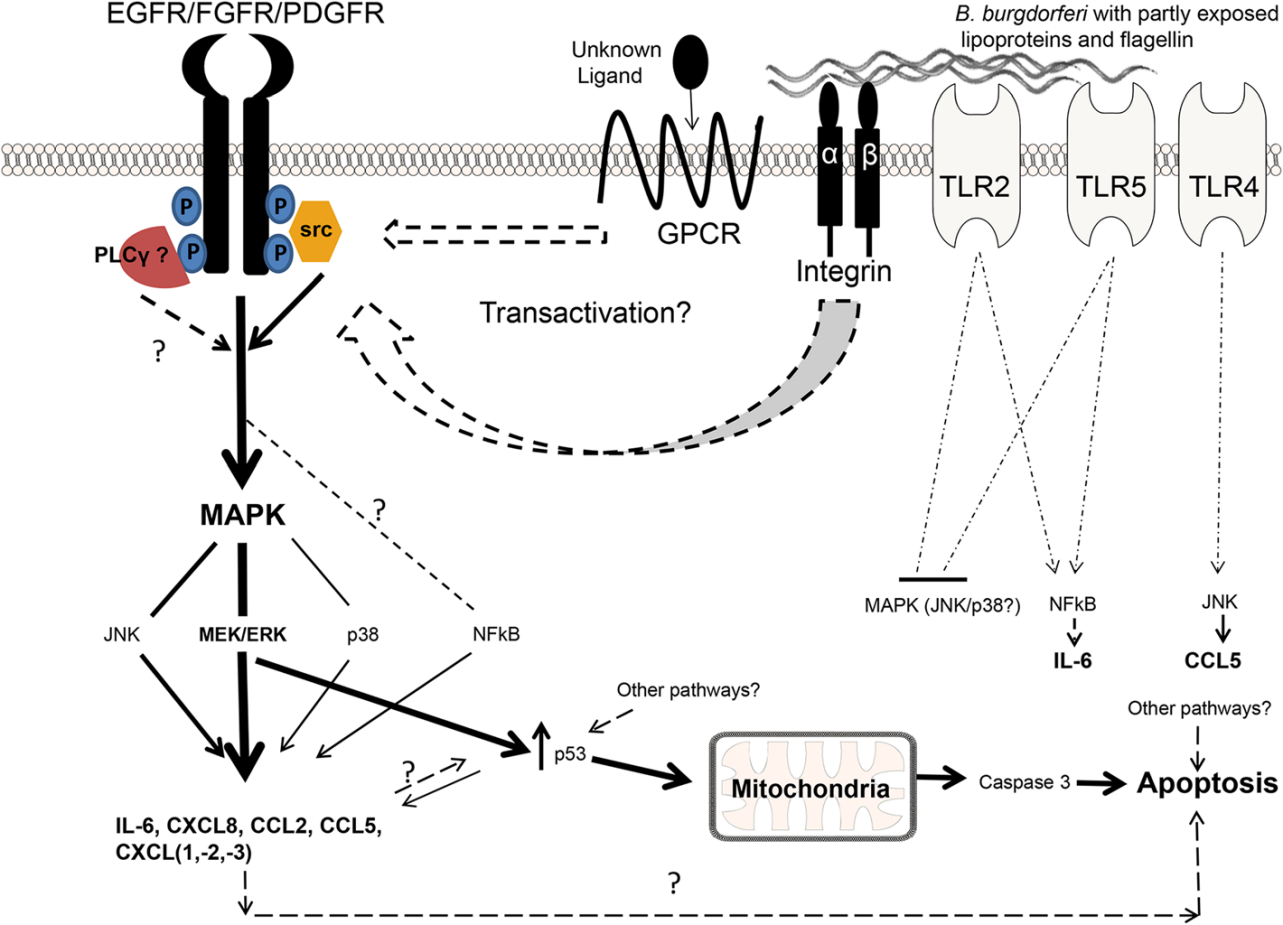
Proposed summary of signaling mechanisms in MO3.13 human oligodendrocytes in response to *B. burgdorferi*. Activation of RTKs (EGFR/FGFR/PDGFR) by ligand-independent mechanisms, as mediated through GPCR or integrins, leads to phosphorylation of the intracellular domains of RTK. This leads to docking of proteins such as src, phospholipase Cγ (PLCγ) and others, which in turn activate downstream pathways like MAPK and MEK/ERK in particular. Significant activation of MEK/ERK, along with contribution from other MAPK and NFkB, leads to production of inflammatory mediators. We have demonstrated previously that MEK/ERK activation upregulates p53 and mitochondrial-mediated apoptosis in oligodendrocytes. The RTKs, for the sake of simplicity, are depicted as a single entity, although they are three separate receptors. Also, for the sake of illustration, they are surface located, although some could be trafficked internally [30]. *B. burgdorferi* can bind integrins, which could transactivate RTKs. It also has well-known ligands to bind to TLR2 or TLR5. Although gram negative, it does not possess a typical LPS (which binds to TLR4) but rather a LOS. However, TLR2, TLR4, and TLR5 play minimal roles in induction of oligodendrocyte inflammatory mediators, as shown in this study. *Thickness of the lines/arrows* indicates signal strength. *Dashed lines/arrows* indicate unknown possibilities, while *dashed and dotted lines/arrows* indicate additional likely signaling mechanisms. Adapted from Parthasarathy and Philipp [7], by permission

We show here that receptors that have no known PAMP associated with *B. burgdorferi* significantly mediate inflammation and apoptosis of MO3.13 oligodendrocytes. These cells do not produce significant EGF or FGF2 upon exposure to *B. burgdorferi* but have not been evaluated for PDGF expression [7]. However, since inhibition of PDGFR did not elicit a significant downregulation of most chemokines/cytokine (Table 4), it is possible that these receptors are transactivated through ligand-independent mechanisms. G-protein-coupled receptors (GPCR) and integrins have both been demonstrated to transactivate RTKs through multiple mechanisms, including activation of src/src family kinases, which in turn can activate RTK intracellular domain [31, 33–36]. *B. burgdorferi* has been shown to bind and activate integrins, while activation of GPCRs would likely occur upon ligand binding of soluble factors induced through as yet unknown receptor/mechanisms [37, 38]. The precise mechanism of their transactivation is currently unknown. A summary of signaling events along with hypothetical activation mechanisms is described in Fig. 3. Please note that this figure is not exhaustive. As an additional point of interest, the profound increase in chemokines and cytokines that occurred after suppressing FGFR alone is also worthy of follow-up studies. While it is possible that this is due to overexpression of FGFR1 in rhabdomyosarcoma cells, as documented in one study [39], nevertheless, it might reveal a novel role in *B. burgdorferi*-mediated pathogenic mechanisms. Finally, all of the experiments involving spirochetes were performed at an MOI of 10:1. This is the concentration that is used in in vitro experiments by most investigators in the field but it is conceivable that at lower MOIs the effects we observed might not be as marked.

## Conclusions

Unlike previous data, the overall immune response from human oligodendrocytes, upon exposure to *B. burgdorferi*, results from activation of primarily three receptor tyrosine kinases, with as yet unknown activation mechanisms. Such activation also contributes to cell death processes, further underscoring the observation that inflammation in the nervous system due to *B. burgdorferi* results in apoptosis of neuroglial cells. The key receptors that were identified in this study may lead to the uncovering of novel pathogenic mechanisms in LNB.

## Abbreviations

CNS: Central nervous system
CCL2: (C-C) motif chemokine ligand 2
CCL5: (C-C) motif chemokine ligand 5
CSF: Cerebrospinal fluid
CXCL8: (C-X-C) motif chemokine ligand 8
CXCL(1,2,3): (C-X-C) motif chemokine ligands 1,2,3
DMEM: Dulbecco’s modified Eagle medium
DRG: Dorsal root ganglia
dUTP: 2′-Deoxyuridine 5′-triphosphate
ELISA: Enzyme-linked immunosorbent assay
EGF: Epidermal growth factor
EGFR: Epidermal growth factor receptor
ERK: Extracellular signal-regulated kinase
FBS: Fetal bovine serum
FGF: Fibroblast growth factor
FGFR: Fibroblast growth factor receptor
GPCR: G-protein-coupled receptor
IL-6: Interleukin 6
LNB: Lyme neuroborreliosis
LOS: Lipooligosaccharide
LPS: Lipopolysaccharide
MAPK: Mitogen-activated protein kinase
MBP: Myelin basic protein
MEK: MAP kinase kinase
MOI: Multiplicity of infection
MTT: 3-(4,5-Dimethylthiazol-2-yl)-2,5-diphenyltetrazolium bromide
NFkB: Nuclear factor κ B
NGS: Normal goat serum
OspA: Outer surface protein A
OxPAPC: Oxidized 1-palmitoyl-2-arachidonoyl-sn-glycero-3-phosphorylcholine
PBS: Phosphate-buffered saline
PDGF: Platelet derived growth factor
PDGFR: Platelet-derived growth factor receptor
PMA: Phorbol myristate acetate
P/S: Penicillin/streptomycin
RNAi: Ribonucleic acid interference
RTK: Receptor tyrosine kinase(s)
siRNA: Small interfering ribonucleic acid
TLR: Toll-like receptor
TUNEL: Terminal deoxynucleotidyl transferase dUTP nick-end labeling
